# Editorial: Structure and function of chloroplasts, Volume III

**DOI:** 10.3389/fpls.2023.1145680

**Published:** 2023-03-01

**Authors:** Hongbo Gao, Alistair J. McCormick, Rebecca L. Roston, Yan Lu

**Affiliations:** ^1^ College of Biological Sciences and Technology, Beijing Forestry University, Beijing, China; ^2^ Institute of Molecular Plant Sciences, School of Biological Sciences, University of Edinburgh, Edinburgh, United Kingdom; ^3^ Department of Biochemistry, University of Nebraska-Lincoln, Lincoln, NE, United States; ^4^ Department of Biological Sciences, Western Michigan University, Kalamazoo, MI, United States

**Keywords:** chloroplast, photosynthesis, envelope, thylakoid, ribosome

Chloroplasts are endosymbiotic organelles derived from cyanobacteria. They have a double envelope membrane, including the outer envelope and the inner envelope. A complex membrane system, thylakoids, exists inside the chloroplast. It is the site of the light-dependent reactions of photosynthesis. The stroma is the main site of the carbon fixation reactions. Although photosynthesis is a very complicated process with many proteins involved, there are many other important processes that occur in chloroplasts, including the regulation of photosynthesis, the biogenesis and maintenance of the structures, carbohydrate, lipid, tetrapyrrole, amino acid, and isoprenoid metabolism, production of some phytohormones, production of specialized metabolites, regulation of redox, and interactions with other parts of the cell (Sabater, 2018). During evolution, most of the cyanobacterial genes were lost and many of them were transferred into the nuclear genome. A majority of chloroplast proteins are nuclear-encoded and possess an N-terminal transit peptide which helps the protein to be targeted into chloroplasts. Chloroplasts have their own highly reduced genome which works coordinately with the nuclear genome for the biogenesis and function of chloroplasts (Liebers et al., 2022). This Research Topic presents studies covering different aspects of chloroplast function, including photosynthesis, biogenesis, structure, and maintenance. These works push the frontiers of chloroplast research further in the field of plant biology.

## Photosynthesis and its regulation

The chloroplast thylakoid protein RUBREDOXIN1 (RBD1) has previously been proposed to play a role in photosystem II (PSII) assembly in Chlamydomonas and *Synechocystis* (Calderon et al., 2013; Garcia-Cerdan et al., 2019; Kiss et al., 2019), but this had not been investigated in land plants. Che et al. examined an Arabidopsis mutant lacking *RBD1*, *rbd1*, and observed a severe reduction in intact PSII complexes, an increased abundance of assembly intermediates, and a reduction in translation of the central D1 subunit. Although newly synthesized mature D1 and precursor D1/D2 could assemble into the PSII reaction center, larger complexes were nearly completely absent. Thus, RBD1 appears to be critical for PSII assembly and may also be involved in the translation of *D1*.

Alternative oxidase (AOX) is responsible for the alternative electron transfer pathway in mitochondria (Juszczuk and Rychter, 2003), while plant plastid terminal oxidase (PTOX) mediates the chloroplast oxygen-consuming respiratory electron transfer pathway (Nawrocki et al., 2015). Wang et al. found that when AOXs are directed to chloroplasts *via* a chloroplast-specific targeting sequence in Arabidopsis, all five AOXs (AOX1a, 1b, 1c, 1d, and AOX2) are able to either partially or fully suppress the variegation phenotype of a *PTOX*-deficient mutant *immutans* indicating that all AOXs could act as a PQH2 oxidase and active PTOX in chloroplasts. The authors also found that native versions of AOX1a, AOX1b and AOX2 were partially dual-localized to chloroplasts, whereas AOX1c and AOX1d are found only in mitochondria. This research revealed the interaction between mitochondria and chloroplasts and shed light on the complex mechanisms of redox control in plant cells.

Li et al. studied the role of the plastidial enzyme ribulose-5-phosphate-3-epimerase (RPE) which plays an important role in the Calvin-Benson-Bassham (CBB) cycle and oxidative pentose phosphate pathways in plants. Using *rpe* knockdown mutants in *Arabidopsis thaliana*, these researchers showed that reduced levels of RPE resulted in decreased leaf CO2 assimilation and photosynthetic electron transport rates under high light levels. Together their findings indicate that RPE may be an additional putative target for increasing flux through the CBB cycle to enhance photosynthesis.

Mn^2+^ is critical for PSII function. It is supplied to the thylakoid lumen by PAM71, a Mn^2+^ transporter and a member of the Uncharacterized Protein Family 0016 (UPF0016, Eisenhut et al., 2018). Although Mn^2+^ transport appears to be a common feature of UPF0016 proteins, little is known about their history. Hoecker et al. used a phylogenetic approach to classify eukaryotic UPF0016 genes into two subgroups. Furthermore, the authors investigated if UPF0016 transporters from different origins could substitute for PAM71, including a cyanobacterial protein MNX, human TMEM165 and an Arabidopsis chloroplast envelope protein CMT1, when directed to the thylakoid membrane in an Arabidopsis *pam71* mutant. In all three cases, the transporters could substitute for PAM71 in a non-native environment, indicating that Mn2+ transport is an ancient feature of the family.

Chlorophyll biosynthesis is catalyzed by the rate-limiting heterotrimeric enzyme, Mg-chelatase. Recent genome sequencing of pea (*Pisum sativum* L.) showed there were two genes of one Mg-chelatase subunit, *PsCHLI1* and *PsCHLI2* (Kreplak et al., 2019). Wu et al. studied the two genes and showed that *PsCHLI1* was more highly expressed than *PsCHLI2* in leaves, that silencing *PsCHLI1* resulted in yellow leaves and reduced chlorophyll content, and that silencing *PsCHLI2* produced no obvious phenotype. The researchers concluded that PsCHLI1 was the essential CHLI subunit for maintaining Mg-chelatase activity, and a potential target for improving photosynthetic efficiency by manipulating Mg-chelatase.

The majority of the light energy is transferred through the linear electron transport (LET) pathway, which includes PSII, cytochrome b6f complex (Cytb6f), photosystem I (PSI) and ferredoxin-NADP reductase (FNR), to ultimately reduce NADP^+^ to NADPH. However, additional pathways, such as a Proton Gradient Regulation5 (PGR5)/PGR5-Like Photosynthetic Phenotype1 (PGRL1)-dependent cyclic electron transport (CET) pathway around PSI, also exist (Joliot and Johnson, 2011). In the minireview of Ma et al., the authors provide an overview on this CET pathway and how it coordinates with other related photosynthesis processes, such as state transition, non-photochemical quenching (NPQ), and the balance of ATP/NADPH, to protect photosystems and chloroplasts during various stress conditions. A deeper understanding of PGR5/PGRL1-CET will be beneficial for the agricultural production.

## Biogenesis and development of chloroplasts

In higher plants, chloroplast development requires coordinated expression of plastid-encoded and nuclear-encoded genes (Liebers et al., 2022). Kong et al. discovered that a novel chloroplast protein, RNA PROCESSING8 (RP8), is required for chloroplast gene expression and chloroplast development in *Arabidopsis thaliana*. Loss-of-function mutation in the *RP8* gene results in reduced accumulation of the mature *rpoA* transcript, which encodes the alpha subunit of the plastid-encoded RNA polymerase (PEP) complex. Consequently, the pale-green *rp8* mutant displays impaired transcription of PEP-dependent genes, such as *psaA, psbA, psbB, petB*, and *rbcL*. Thylakoids are either absent or barely visible in the cotyledons and true leaves of the *rp8* mutant. Therefore, Kong et al. proposed that RP8 is involved in the processing of *rpoA* transcripts.

Ribosome biogenesis is a multistep process that includes the synthesis, processing, and folding of rRNAs, the synthesis, processing, and folding of ribosomal proteins, and finally integration of the ribosomal proteins with the mature rRNAs (Weis et al., 2015). Chen et al. characterized a chloroplast protein CDB1 which is indispensable for chloroplast development through its involvement in chloroplast ribosome assembly. CDB1L, the paralog of CDB1, is localized in both chloroplasts and mitochondria; it may play a similar role during ribosome assembly in both organelles. These findings provide a better understanding of the regulation mechanisms controlling chloroplast development and ribosome assembly in plant organelles.

*Arabidopsis thaliana Ribosomal small subunit methyltransferaseD* (*AtRsmD*) encodes a 16S rRNA methyltransferase in chloroplasts (Ngoc et al., 2021). In the study of Wang et al., the *atrsmd-2* mutant exhibited impaired chloroplast development and reduced photosynthetic efficiency in emerging leaves under normal growth conditions. Amounts of chloroplast-encoded photosynthetic proteins, such as D1, D2, CP43, and CP47, were reduced in the emerging leaves of the *atrsmd-2* mutant, resulting in the decreased accumulation of the photosynthetic super complex. Knockout of the *AtRsmD* gene affected the accumulation of chloroplast rRNAs and chloroplast ribosomal proteins, as well as altered the RNA loading of chloroplast ribosomes in Arabidopsis, with cold stress exacerbating the effect of the mutation on chloroplast development and chloroplast ribosome biogenesis. This work extends our understanding of the significance of chloroplast rRNAs methylation in chloroplast development and photosynthesis.

## Methods for chloroplast research

Chloroplast isolation is a method frequently used in the study of chloroplasts (Fitzpatrick and Keegstra, 2001; Seigneurin-Berny et al., 2008). In many experiments, the intactness of isolated chloroplasts is essential for the validity of conclusions made. However, the intactness of chloroplast envelope was not checked in the many publications, even though this is an essential quality control. An et al. developed a quick and easy method to visualize the intactness of chloroplast envelopes by staining isolated chloroplasts with fluorescent dyes, Rhodamine or Nile red, and then observing the chloroplasts with a fluorescence microscope. Broken chloroplasts and intact chloroplasts can be directly observed. Moreover, the authors have also reported that the middle-layer chloroplasts in Percoll density gradient centrifugation methods may contain mostly broken plastids, a finding that has important practical consequences.

In wild-type plants, chloroplast division proteins are known to form a ring at the division site, with the patterns of these proteins being disordered in several chloroplast division mutants (Wang et al., 2017; Chen et al., 2018; Chen et al., 2019; Liu et al., 2022; Sun et al., 2023). This is often observed *via* immunofluorescence staining (IFS). However, the traditional IFS method uses wax-embedding and sectioning, which is time-consuming and tedious. Wang et al. developed a method that is very simple and fast. They cut leaves into irregular small pieces and performed the IFS directly. The leaf tissue was lysed so that the samples could separate into single cells, which provided a clear view of individual cells. The authors demonstrated the utility of this method by studying the localization of chloroplast division protein FtsZ1 in the wild-type and mutant Arabidopsis and various other plants.

In chloroplasts, stacked thylakoid membranes, grana, are connected by unstacked thylakoid membranes, lamella, forming a complicated membrane network (Kirchhoff, 2019). Thylakoid structure, usually observed *via* electron microscopy, affects the photosynthesis efficiency and is regulated by various developmental and physiological factors. The minireview by Mazur et al. overviews the recent approaches for measuring the ultrastructural features of grana. The authors outline and define structural parameters, such as granum height and diameter, thylakoid thickness, end-membrane length, stacking repeat distance, and granum lateral irregularity, highlighting the basic measurements and related workflows. The paper also discusses how to correctly interpret such data by taking into account the 3D nature of grana stacks projected onto 2D images.

Together, the studies collected here in this special issue represent advances across the topics related to the structure and function of chloroplasts, from biogenesis to regulation, from energy fixation to dissipation, from physical to analysis methods. They will empower future research to delve a little further into the critical questions surrounding chloroplast structure and function.

## Author contributions

All authors listed have made a substantial and intellectual contribution to the work and approved it for publication.
